# Bacteriological profile and antibiogram of blood culture isolates from bloodstream infections in a rural tertiary hospital in Nigeria

**DOI:** 10.4102/ajlm.v11i1.1807

**Published:** 2022-08-24

**Authors:** Oluwalana T. Oyekale, Bola O. Ojo, Adewale T. Olajide, Oluwatoyin I. Oyekale

**Affiliations:** 1 Department of Medical Microbiology and Parasitology, Faculty of Medicine and Health Sciences, Afe Babalola University, Ado Ekiti, Nigeria; 2 Department of Medical Microbiology and Parasitology, Federal Teaching Hospital, Ido-Ekiti, Nigeria; 3 Department of Surgery, Faculty of Medicine and Health Sciences, Afe Babalola University, Ado Ekiti, Nigeria; 4 Department of Surgery, Federal Teaching Hospital, Ido-Ekiti, Nigeria; 5 Department of Radiology, Federal Teaching Hospital, Ido-Ekiti, Nigeria

**Keywords:** antibiotic-resistance, antibiotic sensitivity, bacterial isolates, blood culture, bloodstream infections

## Abstract

**Background:**

Bloodstream infections (BSIs) are a cause of significant morbidity and mortality requiring urgent antibiotic treatment. However, there is widespread antibiotic-resistance from the bacterial causes, necessitating regular surveillance for drug-resistant bacteria and their antibiograms.

**Objective:**

This study isolated and identified various bacterial causes of BSIs, determined their antibiotic susceptibility patterns, and determined the best empirical treatment for cases of BSI in the setting.

**Methods:**

A cross-sectional study was carried out at the Federal Teaching Hospital, Ido-Ekiti, Nigeria between June 2020 and February 2021 on 177 blood culture samples from cases of BSI. Identification of isolated bacteria and antibiotic susceptibility testing of the isolates were carried out following the standard protocol.

**Results:**

Culture positivity in this study was 19.2%. No significant difference was seen in culture positivity between male and female participants (*p* = 0.97). Gram-negative enteric bacteria were predominantly isolated (67.6%), including *Escherichia coli* (29.4%) and *Klebsiella aerogenes* (20.6%). *Staphylococcus aureus* was the most common Gram-positive bacterium isolated (23.5%). Three (37.5%) *S. aureus* isolates were methicillin-resistant. All isolates were sensitive to meropenem, and 97.1% were sensitive to imipenem; other sensitivity patterns were: ceftazidime (85.3%), ciprofloxacin (79.4%), ofloxacin (79.4%), and gentamicin (76.5%). There was low sensitivity to ampicillin (32.4%) and cotrimoxazole (38.2%). All Gram-positive isolates, including methicillin-resistant *S. aureus*, were sensitive to vancomycin.

**Conclusion:**

Regular surveillance of isolate sensitivity patterns, formulation of hospital antibiotic policies based on existing data and compliance with treatment guidelines will promote rational antibiotic use and reduce resistance among bacteria.

## Introduction

Bloodstream infections (BSIs), which range from self-limiting bacteraemia to an outright life-threatening septicaemia, are some of the most common healthcare-associated infections globally.^1^ Bacteraemia is simply described as the presence of viable bacteria in the blood, while septicaemia connotes systemic manifestations caused by bacteria or their toxins in blood. Septicaemia constitutes a significant cause of morbidity and mortality, requiring prompt assessment, diagnosis, and antibiotic treatment.

Bloodstream infections account for up to 9% – 11% of hospital-acquired infections in the developed countries of Europe and the United States, while higher prevalence of up to 19% has been recorded from low- and middle-income countries of the world.^2,3^ A European study had estimated that BSI patients spend an additional 6.0–11.5 days in hospital compared to other patients and the cost of management associated with BSI ranges from $8000 United States dollars (USD) to $56 000 USD.^4^

The risk factors for BSIs include the use of healthcare devices such as: peripheral and central venous catheters on patients; age (elderly patient, neonates); and premorbid medical conditions of patients, such as diabetes mellitus, malignancies, renal failure, burns, and prior hospitalisation.^5^ The mortality rate from bloodstream infections ranges from 4.0% to 41.5% depending on severity, age, sex, and other risk factors. Infections due to antibiotic-resistant strains of bacteria present with a significantly higher morbidity and mortality.^6^

Blood culture remains the gold standard in the laboratory diagnosis and identification of bloodstream pathogens; however, bacteria are not isolated in many cases of BSI.^7^ Blood culture positivity rates among patients with BSI in developing countries range from 9.2% to 44.0%. There is a paucity of data from Nigeria on BSI as a result of a poor surveillance system for the associated pathogens, thus depriving us of any useful antibiotic policy or treatment guidelines for these infections.^8,9^ The few studies carried out in Nigeria on BSIs were mainly among neonates and younger children. Ogunkunle et al., Iregbu et al., and Uzodima et al.*,* all from Nigeria, reported blood culture positivity rates of 19.0%, 22.0% and 35.0%, respectively, among suspected cases of BSI.^10,11,12^

Numerous bacteria have been associated with causation of BSIs including Gram-negative bacteria: *Escherichia coli*, *Pseudomonas* species (spp.), *Klebsiella* spp., *Serratia* spp.*, Salmonella* spp. and *Enterobacter* spp.; and Gram-positive bacteria: *Staphylococcus* spp., *Streptococcus* spp. and *Enterococcus* spp.^13,14^ However, recent findings suggested an upsurge in BSIs caused by multidrug-resistant bacteria, including the members of the Enterobacteriaceae family and other Gram-negative bacteria, such as *Klebsiella* spp., *Pseudomonas* spp., *Acinetobacter* spp. and *Citrobacter* spp., most of which are extended spectrum beta-lactamase (ESBL) producers, and also some Gram-positive bacteria, including methicillin-resistant *Staphylococcus aureus* (MRSA) and the vancomycin-resistant enterococci.^15,16^

The carbapenem antibiotics remain the antibiotic agents of choice in the management of emerging ESBL-producing Gram-negative bacteria, while vancomycin is the mainstay in treatment of MRSA.^17^ However, of particular concern is the recent emergence of increasing resistance of some Gram-negative bacteria to carbapenems through the production of enzymes, carbapenemases.^18^

This trend of multidrug resistance, especially among the Gram-negative bacteria causing BSIs, has created a very serious therapeutic dilemma, especially in the management of intensive care unit patients, since it leads to fewer therapeutic options, use of more expensive drugs, increased hospital stay, and increased morbidity and mortality.^19^

Bearing in mind this trend of antibiotic-resistant bacterial agents of BSIs and the known fact that antibiotic-resistance patterns vary with geographical locations, regular surveillance and documentation of blood culture isolates and their antibiogram is imperative in formulating an antibiotic policy and identifying the best empirical antibiotic therapeutic options for different scenarios of BSIs in each hospital environment.^20^ This will encourage ‘rational use’ of antibiotics and reduce the tendency towards increasing antibiotic-resistance.

There have been random reports of multiple antibiotic-resistant isolates causing a therapeutic dilemma among patients with BSIs in our hospital with occasional attendant case fatalities in the past years. Also, there was no existing antibiotic policy or treatment guideline for BSI management in our centre, as there was poor or non-existent surveillance for these multiple antibiotic-resistant pathogens. These factors necessitated this study.

The objectives of this study are: to isolate and identify different bacterial causes of BSIs; to determine the antibiotic susceptibility patterns of isolated bacteria; and to suggest the best empirical treatment of BSIs in different scenarios in the hospital.

## Methods

### Ethical considerations

Ethical approval (Protocol number: ERC/2020/10/16/431A) for the study was obtained from the Human Research and Ethics Committee, Federal Teaching Hospital, Ido-Ekiti, Nigeria. Written informed consent was also obtained from all participants prior to inclusion in the study. For underage participants, consent was sought and obtained from the parent/guardian. Participants were de-identified by encoding their names with research numbers determined based on the units from which participants were recruited.

### Study design and hospital setting

This cross-sectional study was conducted at the Department of Medical Microbiology and Parasitology of Federal Teaching Hospital, Ido-Ekiti, a rural southwestern Nigerian teaching hospital. The hospital is a tertiary health facility which serves as a referral centre to other primary and secondary healthcare facilities in Ekiti State, Nigeria. It is a 290-bed hospital with many modern facilities for healthcare.

### Study population and sampling method

Using a simple random sampling technique, a total of 177 clinically diagnosed BSI patients were recruited into the study, thus a total of 177 blood culture samples from different hospital units were received and investigated at the medical microbiology laboratory of the hospital between June 2020 and February 2021. Patients’ clinical history and other relevant details were recorded in a predesigned form.

### Blood sample collection and processing

A set of two venous blood samples from two different sites were collected 30 minutes apart from each participant with suspected BSI, following strict aseptic precautions before commencement of antibiotic treatment. Each set consisted of 8 mL – 10 mL of venous blood from adults, 2 mL of venous blood from neonates and 2 mL – 5 mL from other paediatric patients. Blood samples were immediately inoculated into BACT/ALERT® (bioMérieux, Inc., Durham, North Carolina, United States) aerobic blood culture bottles (adult or paediatric bottles, as necessary). These bottles were immediately incubated in a BACT/ALERT^®^ 3D automated blood culture analyser (bioMérieux, Inc., Durham, North Caroline, United States). All BACT/ALERT-positive broths were immediately brought out for subculture onto blood agar (Oxoid, Wade Road, Basingstoke, United Kingdom) and MacConkey agar (Oxoid, Basingstoke, United Kingdom). For those bottles without positive signals, blind subculture onto blood agar and MacConkey agar was done on days 2, 5 and 7 of incubation. The inoculated blood agar and MacConkey agar plates were incubated at 37 ^°^C for 18 hours – 24 hours. Any bacterial growth on agar plates was identified using colonial morphology, gram staining, and conventional biochemical tests using standard laboratory protocols.^21^

Antibiotic susceptibility testing of isolated bacteria was performed on Mueller-Hinton agar (Oxoid, Basingstoke, United Kingdom) using the modified Kirby-Bauer disc diffusion method. The results were interpreted as sensitive or resistant using the guidelines of the Clinical and Laboratory Standard Institute.^22^

The following antibiotics were tested on all identified isolates: imipenem (10 μg), meropenem (10 μg), ampicillin/sulbactam (20/10 μg), gentamicin (10 μg), ciprofloxacin (5 μg), ofloxacin (5 μg), cefriaxone (30 μg), ceftazidime (30 μg), amoxycillin/clavulanate (20/10 μg), cefixime (5 μg), and ampicillin (10 μg). All antibiotic discs were from Oxoid^TM^ (Oxoid, Wade Road, Basingstoke, United Kingdom). All Gram-negative bacteria isolates were tested for ESBL production by the double disc synergy test using ceftazidime (30 μg) and ceftazidime/clavulanate (30/10 μg) discs, while the cefoxitin disc diffusion method was used to identify MRSA.^22^ All *S. aureus* isolated, including MRSA and coagulase-negative *S. aureus* (CoNS), were tested against vancomycin using ETEST® (bioMérieux, Inc., Durham, North Carolina, United States).

### Inclusion and exclusion criteria

All blood samples of patients with suspected BSI without a history of antibiotic medication prior to sample collection were included, while blood samples of BSI patients with a history of antibiotic medication prior to sample collection were excluded.

### Data entry and analysis

Data were entered into Microsoft Excel 2017 (Microsoft Corporation, Redmond, Washington, United States) and data analysis was done using the Statistical Package for Social Sciences version 20.0 (IBM Corp., Armonk, New York, United States). Results were presented in tables and expressed as frequencies and percentages. Association between variables was tested using Chi-square and/or Fisher’s exact tests, as appropriate. Statistical significance was accepted at *p* ≤ 0.05.

## Results

The age range of participants was 4 days to 87 years (mean: 23.29 ± 26.58 years) (Table 1). Of the 177 suspected cases of sepsis and other bloodstream-related infections that were investigated, 102 (57.6%) of the patients were male, and 75 (42.4%) were female. There were 27 (26.5%) male patients and 15 (20.0%) female patients below the age of one month (neonates), and 15 (14.7%) male patients and 23 (30.7%) female patients older than 50 years.

**TABLE 1:** Age distribution of bloodstream infection study participants in relation to gender at the Federal Teaching Hospital, Ido-Ekiti, Nigeria, between June 2020 and February 2021.

Age group	Male		Female		Total	
	*n*	%	*n*	%	*n*	%
< 1 month	27	26.5	15	20.0	42	23.7
1 month – 5 years	23	22.5	12	16.0	35	19.8
6 years – 17 years	21	20.6	10	13.3	31	17.5
18 years – 50 years	16	15.7	15	20.0	31	17.5
> 50 years	15	14.7	23	30.7	38	21.5

**Total**	**102**	**57.6**	**75**	**42.4**	**177**	**100.0**

Mean = 23.29; Variance = 706.3; Standard deviation = 26.58.

Only 34 (19.2%) of the total blood culture broths were culture-positive for bacteria (Table 2). Culture positivity was highest among neonates (aged < 1 month; 23.8%) and lowest among patients aged 6–17 years (12.9%). Culture positivity was higher among female patients (20.0%) compared to male patients (9.5%) aged 6–17 years, but no significant difference was seen (*p* = 0.57). No significant difference was seen in the overall isolation rate between male and female patients (*p* = 0.97).

**TABLE 2:** Frequency of culture-positive broths by age group among bloodstream infection cases at the Federal Teaching Hospital, Ido-Ekiti, Nigeria, between June 2020 and February 2021.

Age group	Male		Female		Total		χ2 value	*p*
	*n*	%	*n*	%	*n*	%		
< 1 month	7	25.9	3	20.0	10	23.8	0.19	1.00^†^
1 month – 5 years	4	17.4	3	25.0	7	20.0	0.01	0.67^†^
6 years – 17 years	2	9.5	2	20.0	4	12.9	0.06	0.57^†^
18 years – 50 years	2	12.5	3	20.0	5	16.1	0.01	0.65^†^
> 50 years	4	26.7	4	17.4	8	21.1	0.08	0.69^†^

**Total**	**19**	**18.6**	**15**	**20.0**	**34**	**19.2**	**0.05**	**0.97**

† , Fisher’s exact test.

The most commonly isolated bacteria were *E. coli* (10/34, 29.4%), *S. aureus* (8/34, 23.5%), and *K. aerogenes* (7/34, 20.6%) (Table 3). Gram-negative bacteria species were the most commonly isolated (23/34 isolates, 67.6%) (Table 4). Gram-negative bacteria were isolated at a higher rate among neonates < 1 month (8/42 neonates, 19.0%) compared to other participants older than one month (15/135 participants, 11.1%) but no significant difference was seen in the rate of isolation (*p* = 0.18). The isolation rate was higher among neonates (10/42 neonates, 23.8%) compared to other participants older than one month (24/135 participants, 17.8%), but no significant difference was found in the isolation rate (*p* = 0.39).

**TABLE 3:** Frequency of isolates from culture-positive broths among bloodstream infection cases by age group at the Federal Teaching Hospital, Ido-Ekiti, Nigeria, between June 2020 and February 2021.

S/N	Isolates	< 1 month (*n* = 42)		1 month – 5 years		6 – 17 years (*n* = 31)		18 – 50 years (*n* = 31)		> 50 years (*n* = 38)		Total (*n* = 177)	
		*n*	%	*n*	%	*n*	%	*n*	%	*n*	%	*n*	%
1	*Escherichia coli*	5	11.9	1	2.9	0	0.0	1	3.2	3	7.9	10	29.4
2	*Staphylococcus aureus*	1	2.4	2	5.7	1	3.2	2	6.5	2	5.3	8	23.5
3	*Klebsiella aerogenes*	2	4.8	2	5.7	1	3.2	0	0.0	2	5.3	7	20.6
4	CoNS	1	2.4	0	0.0	1	3.2	1	3.2	0	0.0	3	8.8
5	*Pseudomonas aeruginosa*	0	0.0	1	2.9	1	3.2	1	3.2	0	0.0	3	8.8
6	*Proteus vulgaris*	1	2.4	1	2.9	0	0.0	0	0.0	0	0.0	2	5.9
7	*Proteus mirabilis*	0	0.0	0	0.0	0	0.0	0	0.0	1	2.6	1	2.9

**Total**		**10**	**23.8**	**7**	**20.0**	**4**	**12.9**	**5**	**16.1**	**8**	**21.1**	**34**	**100.0**

S/N, serial number; CoNS, Coagulase-negative *Staphylococcus aureus.*

**TABLE 4:** Comparison of Gram-positive and Gram-negative isolates from bloodstream infection cases between neonates and other participants at the Federal Teaching Hospital, Ido-Ekiti , Nigeria, between June 2020 and February 2021.

Outcome	Bacterial isolate	< 1 month		Others (> 1 month)		Total positive		Total		*χ*2 value	*p*
		*n*	%	*n*	%	*n*	%	*n*	%		
Positive growth	Gram-negative	8	19.0	15	11.1	23	67.6	23	13.0	1.78	0.18
	Gram-positive	2	4.8	9	6.7	11	32.4	11	6.2	0.01	1.0^†^
	Total isolate	10	23.8	24	17.8	34	100.0	34	19.2	0.75	0.39
No growth	No isolate	32	76.2	111	82.2	-	-	143	80.8	-	-

**Total**	**Total cultured**	**42**	**23.7**	**135**	**76.3**	**-**	**-**	**177**	**100.0**	**-**	**-**

† , Fisher’s exact test.

All (100.0%) of the isolates were sensitive to meropenem, 33/34 isolates (97.1%) were sensitive to imipenem, 29/34 isolates (85.3%) were sensitive to ceftazidime, 27/34 isolates (79.4%) were sensitive to ciprofloxacin and ofloxacin, and 26/34 isolates (76.5%) were sensitive to gentamicin (Table 5). Very poor sensitivity was observed for ampicillin (11/34 isolates, 32.4%) and cotrimoxazole (13/34 isolates, 38.2%). None of the Gram-negative enteric bacteria isolated were ESBL producers. Three (37.5%) of the eight *S. aureus* isolated were MRSA. All of the isolated CoNS (3/3 isolates, 100%) and *S. aureus* (8/8 isolates, 100%), including the MRSA, were sensitive to vancomycin.

**TABLE 5:** Antibiotic susceptibility pattern of isolates from bloodstream infection cases at the Federal Teaching Hospital, Ido-Ekiti, Nigeria, between June 2020 and February 2021.

Antibiotics	Number and percentage of sensitive bacteria															
	*Escherichia coli* (*n* = 10)		*Staphylococcus aureus* (*n* = 8)		*Klebsiella aerogenes* (*n* = 7)		CoNS (*n* = 3)		*Pseudomonas aeruginosa* (*n* = 3)		*Proteus vulgaris* (*n* = 2)		*Proteus mirabilis (n* = 1)		Total (*n* = 34)	
	*n*	%	*n*	%	*n*	%	*n*	%	*n*	%	*n*	%	*n*	%	*n*	%
Ampicillin	5	50.0	3	37.5	0	0.0	1	33.3	0	0.0	1	50.0	1	100.0	11	32.4
Cefixime	7	70.0	6	75.0	5	71.4	2	66.7	1	33.3	2	100.0	1	100.0	24	70.6
Amoxycillin/Clavulanate	7	70.0	5	62.5	4	57.1	2	66.7	1	33.3	2	100.0	1	100.0	22	64.7
Ampicillin/Sulbactam	7	70.0	5	62.5	5	71.4	2	66.7	1	33.3	2	100.0	1	100.0	23	67.6
Imipenem	10	100.0	7	87.5	7	100.0	3	100.0	3	100.0	2	100.0	1	100.0	33	97.1
Meropenem	10	100.0	8	100.0	7	100.0	3	100.0	3	100.0	2	100.0	1	100.0	34	100.0
Ceftriaxone	7	70.0	5	62.5	5	71.4	2	66.7	1	33.3	2	100.0	1	100.0	23	67.6
Ciprofloxacin	8	80.0	6	75.0	5	71.4	3	100.0	2	66.7	2	100.0	1	100.0	27	79.4
Ofloxacin	8	80.0	6	75.0	5	71.4	3	100.0	2	66.7	2	100/0	1	100.0	27	79.4
Gentamicin	8	80.0	5	62.5	6	85.7	2	66.7	2	66.7	2	100.0	1	100.0	26	76.5
Ceftazidime	9	90.0	5	62.5	7	100.0	2	66.7	3	100.0	2	100.0	1	100.0	29	85.3
Cotrimoxazole	4	40.0	3	37.5	4	57.1	1	33.3	0	0.0	1	50.0	0	0.0	13	38.2
Cefoxitin	-	-	5	62.5	-	-	-	-	-	-	-	-	-	-	-	-
Vancomycin	-	-	8	100.0	-	-	3	100.0	-	-	-	-	-	-	-	-

CoNS, Coagulase-negative *Staphylococcus aureus.*

## Discussion

The low culture positivity rate of 19.2% seen in this study is similar to findings in some previous studies. Deku et al. reported a culture positivity rate of 13.1% in Ghana and Gupta et al. reported 16.5% in North India.^23,24^ Lower rates have been reported in other studies: Khanal et al. and Gohel et al. reported a culture positivity rate of 10.3% and 9.2%, respectively, from BSI cases in India.^8,25^ A higher rate of 44.0% has been reported by Khanal et al. in another prospective study on patients with infective endocarditis in India.^9^ Most similar studies conducted in Nigeria and Africa focused mainly on neonatal and childhood BSIs, and data on adult BSIs is scanty. Of those studies on neonatal BSIs, some have reported similar culture positivity rates to the 23.8% seen among neonates in this study: Ogunkunle et al. (19.0%) and Iregbu et al. (22.0%), both in Nigeria, while higher isolation rates were reported by Sorsa in Ethiopia (29.3%), Uzodimma et al. in Nigeria (35.0%) and El-Din et al. in Egypt (40.7%).^10,11,12,26,27^ Factors determining rate of culture positivity include the methods or technique used in isolating bacteria, volume and the number of blood samples collected for the culture; also in addition, prior use of antibiotics before sample collection will affect the likelihood of culture positivity.^28^ In this study, adequate volumes of blood samples were collected before the commencement of antibiotic treatment, and patients with history of antibiotic medication prior to sample collection were excluded from the study, yet, prior self-medication at home or use of antibiotics at peripheral health facilities before transfer to our centre could not be totally ruled out. This coupled with the fact that some of these patients clinically diagnosed as BSI may actually have been suffering from other conditions mimicking BSI (and not BSI), thus accounting for the low culture positivity seen.

Gram-negative enteric bacteria (67.6%) were the predominant isolates in this study, of which *E. coli* (29.4%) was the most commonly isolated. *Staphylococcus aureus* (23.5%) and CoNS (8.8%) were the only Gram-positive bacteria isolated. Similar findings have been reported by earlier studies where Gram-negative bacteria were the predominant isolates from cases of BSI.^24,28^ However, some studies have reported *S. aureus* or CoNS as the most commonly isolated bacterial pathogen from cases of BSI.^15,23,25,29,30,31^ Generally, there is wide variability in the pathogens isolated from cases of BSI in different settings. Gram-positive bacteria were the most common cause of sepsis prior to the advent of antibiotics in the 1950s, but Gram-negative bacteria became the most predominant after the introduction of antibiotics from the 1960s to 1980s. However, from the 1980s, Gram-positive bacteria, most commonly *Staphylococcus* spp., were thought to cause more than 50% of cases of sepsis.^32,33^ In hospital patients, there is a higher chance of hospital-selected Gram-negative bacteria causing BSIs due to the instruments and procedures carried out on these patients. This may account for the preponderance of Gram-negative bacteria isolates in this study.

Predominant isolation of Gram-negative enteric bacteria among neonates in this study contrasted with some previous studies on neonatal BSIs: Ogunkunle et al., Sorsa and El-Din et al. all reported Gram-positive bacteria as the most commonly isolated pathogens from cases of neonatal BSIs.^10,26,27^ This contrasting result may be because of the small number of neonates examined in this study compared to those other studies that focused mainly on neonates. Iregbu et al., however, reported isolation of Gram-positive and Gram-negative bacteria in equal proportion from cases of neonatal BSIs in Abuja, Nigeria.^11^

All of the CoNS and *S. aureus*, including the three MRSA isolated in this study, were sensitive to vancomycin. Resistance of CoNS and MRSA to vancomycin is of immense challenge to the treatment of diseases caused by these strains because of associated persistent infections, vancomycin treatment failure and a generally poor clinical outcome. All of the isolates in this study were sensitive to meropenem, and 97.1% were sensitive to imipenem. These carbapenems represent the last resort, in antibiotic treatment, for most facilities in the less-developed world, where newer antibiotics are out of reach of the majority, especially for ESBL-producing Gram-negative bacteria.^17^ Favourable sensitivity patterns were also seen in this study to other antibiotics, including ceftazidime (85.3%), ofloxacin (79.4%), ciprofloxacin (79.4%), and gentamicin (76.5%). The poor sensitivity pattern seen with these isolates to cotrimoxazole (32.4%), ampicillin (38.2%), amoxycillin-clavulanate (64.7%), ampicillin-sulbactam (67.6%) and ceftriaxone (67.6%) is undoubtedly a result of irrational antibiotic use. Some of these drugs are regularly abused by patients even without prescription while others are indiscriminately used in our health facilities, resulting in the generation of resistance to these antibiotics. Formulation of antibiotic policy on BSIs from this data and compliance with treatment guidelines are of paramount importance, not only to save patients’ lives, but also to reduce further resistance generated by bacteria to more antibiotics.

For the empirical treatment of BSIs in this setting, a combination of ceftazidime with gentamicin is recommended in children less than 16 years of age, while a combination of ciprofloxacin/ofloxacin with gentamicin is recommended in the older age-groups. These three antibiotics demonstrated favourable sensitivity patterns against most bacteria isolated in this study; their combination in treatment of BSIs will not only produce a synergistic effect, but will also reduce the rate at which isolates develop resistance to individual antibiotics. Meropenem and imipenem should be reserved for cases not amenable to the aforementioned combination therapy. It is advisable that meropenem or imipenem should also be used in combination with other classes of antibiotics with a good sensitivity profile to the isolated pathogen, such as gentamicin, to reduce the speed at which bacteria generate resistance to these valuable drugs. In cases of BSI due to MRSA, vancomycin is the recommended treatment of choice in this setting.

### Limitations

The method of antibiotic sensitivity testing employed for all antibiotics (except vancomycin) tested against isolates in this study was limited to disc diffusion sensitivity testing. Additional determination of the minimum inhibitory concentration of the different antibiotics which would have supplied quantitative data on the susceptibility pattern of isolates to these antibiotics was desired, but the study was limited by supply of funds. Similarly, due to limited funding, we were unable to carry out the molecular characterisation of the resistant genes among the MRSA isolated.

Moreover, this study involved only our centre, which may have limited the relevance of data generated as regards antibiotic policy formulation and determination of treatment guideline for managing BSIs throughout Nigeria and other African countries, a multicentre study is desirable in future.

## Conclusion

Gram-negative bacteria were predominantly isolated from cases of BSI. Isolates demonstrated good sensitivity to meropenem, imipenem, ceftazidime, ciprofloxacin/ofloxacin, and gentamicin. Regular surveillance of isolate sensitivity patterns, formulation of hospital antibiotic policies based on existing data, and compliance with treatment guidelines will promote rational antibiotic use and reduce resistance generation among bacteria.
